# Technical assessment of a novel vertical CT system for upright radiotherapy simulation and treatment planning

**DOI:** 10.1002/mp.70312

**Published:** 2026-02-13

**Authors:** Jordan M. Slagowski, Yuhao Yan, Jessica R. Miller, John W. Hayes, Carson A. Hoffman, Minglei Kang, Carri K. Glide‐Hurst

**Affiliations:** ^1^ Department of Human Oncology University of Wisconsin – Madison Madison Wisconsin USA; ^2^ Department of Medical Physics University of Wisconsin – Madison Madison Wisconsin USA; ^3^ Leo Cancer Care, Inc. Middleton Wisconsin USA

**Keywords:** gantry‐less particle therapy, upright CT, upright radiotherapy

## Abstract

**Background:**

Upright patient positioning may provide anatomical advantages and more cost‐effective proton therapy using a fixed beamline.

**Purpose:**

To characterize image quality, imaging dose, and dose calculation accuracy for an upright CT scanner with a six‐degree‐of‐freedom patient positioning system.

**Methods:**

CT imaging dose (CTDI_vol_) was measured at 120 kVp for head and thorax protocols. Image quality was evaluated using an ACR‐464 phantom. Mean CT number accuracy was assessed within inserts of known material, and uniformity as the difference in values at the center and periphery of uniform phantoms. High‐contrast resolution was assessed by visible line pairs and modulation transfer function (MTF). Low‐contrast performance was quantified by contrast‐to‐noise‐ratio (CNR). Spatial integrity was evaluated between fiducials 100 mm apart. Hounsfield unit to mass density and stopping‐power‐ratio calibrations were performed. Proton and photon plans were optimized on upright CT scans of a thorax phantom in heterogeneous and homogeneous regions. Dose was forward computed on a registered recumbent CT scan and agreement evaluated using 3D gamma analysis.

**Results:**

CTDI_vol_ was 23.5 ± 0.02 mGy for the 16 cm head and 10.1 ± 0.01 mGy for the 32 cm body phantoms. Mean CT numbers (HU) were within the expected range for water (1.7) and acrylic (120.8). CT numbers were slightly [5–27 HU] out‐of‐range for air (−950.4), polyethylene (−78.8), and bone (823.0). Image uniformity was 20.2 HU and 35.0 HU for 20 and 48 cm diameter phantoms, respectively. Eight high‐contrast line pairs were visualized. The MTF equaled 4.4 cm^−1^ at 50% and 7.1 cm^−1^ at 10%. The median CNR was 0.93, below the ≥1.0 tolerance. Spatial integrity was ≤0.36 mm. Gamma pass rates were ≥99.8% for photon and ≥90.6% for proton plans with 1%/1 mm criteria, and ≥98.0% for all plans with 3%/2 mm criteria.

**Conclusion:**

Upright CT image quality and dose calculation accuracy are acceptable for photon and proton radiotherapy.

## 1 INTRODUCTION

For more than half a century, nearly all radiation therapy (RT) has been delivered to patients in a recumbent position using a rotating gantry to deliver treatment beams from multiple angles.^1^ However, emerging evidence suggests that upright patient positioning (e.g. seated, perched, or standing) could provide anatomical advantages while also improving patient comfort.^1^, ^2^ By replacing a large (80–120 ton) rotating gantry with a fixed proton beamline and an upright rotating patient positioner, treatment facilities can decrease initial construction, radiation shielding, and ongoing maintenance costs, to make dosimetrically superior charged particle therapies more accessible and economically viable.^3^, ^4^

Despite the successful development of upright patient positioners,^5^, ^6^ the anticipated benefits of upright RT have yet to be fully realized, largely due to the lack of accessible vertical CT imaging systems necessary for anatomical assessment, treatment planning, and patient alignment. Two vertical CT systems integrated with upright patient positioners for radiotherapy applications are now commercially available. P‐Cure Ltd. (Shilat, Israel) developed the Patient Robotic Positioning and Imaging System (P‐ARTIS) which combines a chair mounted to a six‐degree‐of‐freedom robotic arm with a vertical 4DCT imaging system.^7^ Leo Cancer Care, Inc. (Middleton, WI, USA) has developed Marie®, designed specifically for upright radiation therapy, which combines a patient positioner fully integrated with a vertical fan beam CT scanner.^8^

This study presents the first technical characterization of the Marie® upright CT scanner. System design, imaging protocols, and dose measurements are described for the first time. Image quality was evaluated against ACR accreditation standards for low‐ and high‐contrast resolution, uniformity, spatial integrity, and CT number accuracy. CT number to mass‐density and stopping‐power‐ratio (SPR) calibrations were performed. Photon and proton dose calculations on upright CT images were compared with conventional recumbent CT data as an initial step toward clinical implementation.

## 2 METHODS

### 2.1 Upright CT system overview

**FIGURE 1:**
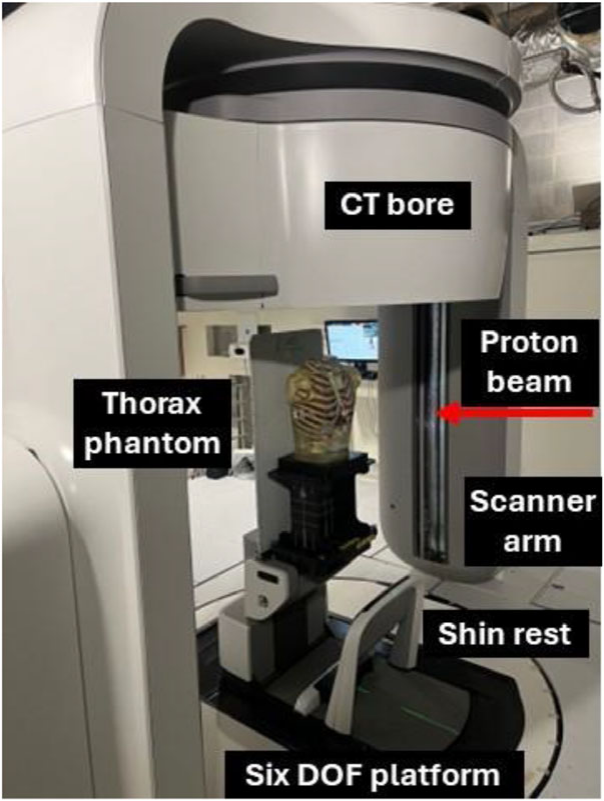
Upright CT system for radiation therapy.

The Marie® system (Leo Cancer Care, Middleton, WI, USA), shown in Figure 1 combines a 32‐slice helical CT scanner with an upright patient positioner. The CT bore is mounted to two scanner arms containing counterweights that enable upright patient imaging with tilt angles up to +/−15°.^8^ X‐rays produced by a rotating anode tube with primary and secondary tungsten collimators pass through a titanium bowtie filter and a detector side anti‐scatter grid with tungsten septa. Image reconstruction is performed by filtered backprojection. System parameters are summarized in Table 1.

**TABLE 1:** Upright CT system parameters.

CT System parameters	
CT bore diameter	85 cm
Vertical travel range	130 cm
X‐ray tube kVp	120
mA	10–250
Rotation time (360°)	1.0 s
Scintillator type	Gadolinium Oxysulfide
Beam width at isocenter	19.3 mm
Reconstruction FOV	16–62.3 cm
Reconstruction filters	Ram‐Lak, Shepp‐Logan
Stored bit depth	16‐bit

### 2.2 CT imaging protocols

Acquisition parameters for all CT imaging performed in this study are summarized in Table 2. Vertical travel speed is determined by the selected pitch. The system travels at 6.66 cm/s for scout imaging and at 19.30 cm/s for a helical pitch of 1.0. The *Thorax UW* protocol was used for treatment planning and *ACR QA* for image quality evaluation. Automatic exposure control (AEC) was set off for all protocols.

**TABLE 2:** Upright CT imaging protocols.

Protocol	Energy [kV]	Exposure [mA]	Pitch	Filter	FOV diameter [cm]	Slice thickness [mm]
Thorax vendor default	120	250	1.0	Ram‐Lak	57.0	2.0
Thorax UW	120	250	1.0	Ram‐Lak	50.0	2.0
Head Hi‐Res adult	120	250	1.0	Ram‐Lak	30.0	1.0
ACR QA	120	250	1.0	Ram‐Lak	21.0	1.0

### 2.3 CT imaging dose

Upright CT imaging dose was measured in terms of CTDI_vol_ using a 100‐mm‐long pencil ionization chamber (Accu‐Dose+, Radcal, Monrovia, CA, USA) with an accredited calibration for 16 cm diameter head and 32 cm body CTDI phantoms. CT imaging was performed at 120 kVp, 200 mA, 32 x 0.605 mm collimation width, and a 1.0 s rotation time. As the upright CT operates clinically in a helical scan mode, to achieve the axial mode required for CTDI measurements, phantom measurements were performed with the CT ring parked at the top of the scanner arms in an engineering mode to enable axial scanning. Three measurements were performed and averaged for each of the center and four peripheral holes. Weighted CTDI was converted to CTDI_vol_ with a pitch value of 1.0. The measured CTDI_vol_ was scaled by mAs for the clinical protocols and compared to values from published recumbent CT simulation protocols.^9^, ^10^

### 2.4 CT image quality evaluation

Upright CT image quality was assessed using an ACR Model 464 CT accreditation phantom following guidelines for diagnostic CT quality control and scanner accreditation.^11^ Image quality tolerances are specified in Tables 3 and 4. The phantom was scanned on seven separate instances over a 54‐day period and the standard deviation (σ) of quality metrics is reported.

**TABLE 3:** Upright CT image quality metrics.

Metric	Median [Min, Max]	σ	Tolerance
Uniformity (HU)	20.2 [18.9, 20.3]	0.52	≤7.0
CNR	0.93 [0.81, 1.10]	0.10	≥1.0
Spatial integrity (mm)	0.23 [0.15, 0.36]	0.07	≤1.0^a^
50% MTF (lp/cm)	4.4 [3.8, 4.7]	0.29	N/A
10% MTF (lp/cm)	7.1 [6.6, 7.2]	0.21	N/A
Visible lp/cm	8.0 [8.0, 8.0]	0.0	≥6

^a^ Tolerance per AAPM TG‐66.^14^ ^.^

**TABLE 4:** Upright CT number accuracy. Median, minimum, and maximum values across seven measurements are reported.

Material	Median [Min, Max] (HU)	σ (HU)	ACR range (HU)
Air	−950.4 [−952.6, −949.5]	1.0	[−1005, −970]
Polyethylene	−78.8 [‐83.3, −75.9]	2.3	[−107, −84]
Water	1.7 [−0.2, 5.4]	1.9	[−7, 7]
Acrylic	120.8 [119.6, 124.0]	1.5	[110, 135]
Bone	823.0 [819.5, 830.2]	3.6	[850, 970]

**FIGURE 2:**
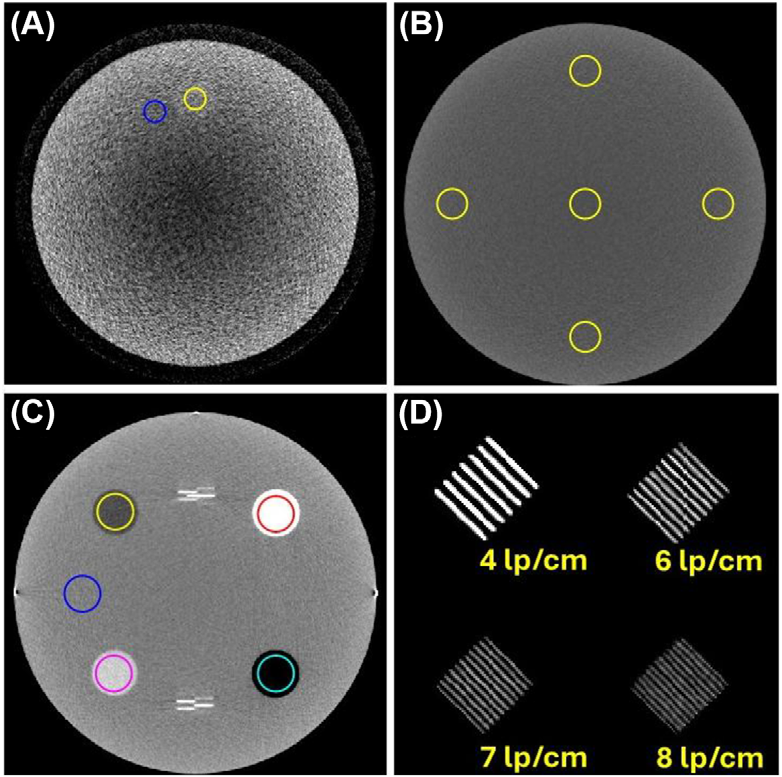
Upright CT images of the ACR phantom are shown for (A) low contrast resolution, display settings [50 HU, 150 HU], (B) uniformity [−200 HU, 400 HU], (C) HU accuracy [−200 HU, 200 HU] and (D) high contrast line pairs [900 HU, 1300 HU].

Low‐contrast performance was measured in terms of the contrast‐to‐noise ratio: $CNR=\left(\mu_{target}-\mu_{background}\right)/\sigma_{background}$. The mean ($\mu_{background}$) and standard deviation ($\sigma_{background}$) of image values in a background region‐of‐interest (ROI) were measured. CT number accuracy was evaluated for material inserts including air, polyethylene, water, acrylic, and bone equivalent Teflon. Image uniformity was quantified as the difference in mean image values between an ROI at the center of the ACR phantom and four peripheral ROIs.^11^ In addition to measurements within the 20 cm diameter ACR‐464 phantom, a 48 cm diameter Helios IQ Cal 48 Poly phantom (Model 2144721‐2, GE Healthcare) was also imaged to evaluate uniformity over a larger field‐of‐view (FOV). ROI placement for all tests is outlined in Figure 2.

To assess high contrast spatial resolution, the radial modulation transfer function (MTF) was derived from the uniformity module of the ACR‐464 phantom.^12^ Spatial frequencies (cm^−1^) corresponding to MTF values of 0.5 and 0.1 were reported to characterize the maximum detectable spatial frequency. The maximum number of visible line pairs per cm (lp/cm) was recorded by a qualified medical physicist. Spatial integrity was assessed by measuring the distance between two fiducial markers separated by 100 mm in the phantom.^13^, ^14^

### 2.5 CT number to density and SPR

CT number to mass density and SPR calibrations were performed to enable photon and proton dose calculations, respectively, within the RayStation 2024A SP3 (RaySearch Laboratories AB, Stockholm, Sweden) treatment planning system (TPS). A 40 cm by 30 cm Gammex Advanced Electron Density Phantom (Gammex, Middleton, WI, USA) was scanned using the upright Marie® CT scanner with the *Thorax UW* protocol and a conventional SOMATOM Definition CT (Siemens Healthineers, Erlangen, Germany) scanner at matched tube voltage and mAs. The phantom was scanned following consensus guidelines.^15^ Mean CT numbers were extracted from cylindrical ROIs within the inserted rods,^16^ and a Hounsfield look‐up table (HLUT) to SPR was generated using the consensus guideline software.^15^ CT number linearity was assessed by computing the coefficient of determination (R^2^) from a linear regression of upright versus recumbent CT numbers (HU) at matched insert densities.

### 2.6 Photon and proton dose calculations

The feasibility of using upright CT for photon and proton treatment planning was evaluated using an anthropomorphic thorax phantom (RSD‐111T, Radiology Support Devices, USA) in a controlled, deformation free setting. The phantom was scanned on the upright CT scanner with the *Thorax UW* protocol and on a recumbent CT with matched acquisition parameters (FOV = 50 cm, voxel size = 0.98 mm, slice thickness = 2.0 mm).

Planning target volumes (PTV) mimicking a lung tumor, spinal metastasis, and liver tumor, along with organs‐at‐risk (OAR), were contoured on the upright CT. The recumbent CT dataset was rigidly registered to the upright CT scan and contours were transferred. Treatment plans were optimized on the upright CT in RayStation using prescription doses and planning objectives summarized in Supplemental Table S1.^17^, ^18^, ^19^, ^20^ Photon plans used VMAT for lung and spine and 3D conformal for liver with a collapsed cone v5.9 Varian TrueBeam model. Proton plans used Hitachi PROBEAT pencil beam scanning with robust optimization (± 3.5% range, ± 5 mm setup) and Monte Carlo v5.6 (0.1% statistical uncertainty).

Optimized plans were re‐computed on the registered recumbent CT using the scanner‐specific HLUTs with a 2.0 mm isotropic dose grid. Dosimetric agreement was evaluated by voxel wise percent differences and 3D gamma analysis with global normalization, 1%/1 mm, 2%/2 mm, and 3%/2 mm criteria per TG‐218,^21^ with a 10% dose threshold using PyMedPhys software.^22^, ^23^

## 3 RESULTS

### 3.1 CT imaging dose

CTDI_vol_ was 18.8 ± 0.02 mGy and 8.1 ± 0.01 mGy for 16 and 32 cm phantoms at 200 mAs. CTDI_vol_ was 23.5 ± 0.02 mGy and 10.1 ± 0.01 mGy for the *Head Hi‐Res Adult* and *Thorax Vendor Default* protocols.

### 3.2 CT image quality evaluation

Upright CT images of the ACR‐464 phantom are presented in Figure 2 for each module. Image quality metrics are summarized in Tables 3 and 4.

#### 3.2.1 Low‐contrast performance

CNR (Table 3) ranged from 0.81 to 1.10 with a median value of 0.93, slightly below the ≥1.0 tolerance. Qualitatively, the 25 mm rod was visible (Figure 2A). None of the 6 mm rods could be visualized regardless of the window and level setting for low‐contrast detectability.

#### 3.2.2 CT number accuracy

Measured CT numbers for each of the material inserts are summarized in Table 4. Mean CT numbers were within the expected range for water and acrylic. CT numbers were slightly [5–27 HU] out‐of‐range for air, polyethylene, and bone.

#### 3.2.3 Image uniformity

Median uniformity was 20.2 HU (Table 3) which exceeded the tolerance of ≤7.0 HU. No major artifacts such as rings were observed in Figures S1, S2, S3, and S4. Uniformity was 35.0 HU in the 48 cm diameter Helios IQ Cal 48 Poly phantom (Figure S1).

#### 3.2.4 High‐contrast spatial resolution

**FIGURE 3:**
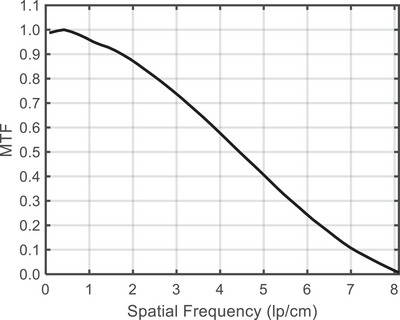
Modulation transfer function.

Figure 2D presents magnified images of a subset of the high‐contrast line‐pairs within the phantom. The maximum number of line‐pairs visualized was 8 line‐pairs per cm (lp/cm) which satisfied the ACR tolerance of ≥6 lp/cm. Figure 3 shows the MTF. The MTF was reduced to 4.4 cm^−1^ at 0.5 and 7.1 cm^−1^ at 0.1 (Table 3, median values).

#### 3.2.5 Spatial integrity

The median geometric error equaled 0.23 mm (Table 3). All measured errors were less than or equal to 0.36 mm thereby satisfying the AAPM Task Group 66 tolerance of ≤1 mm.^14^

### 3.3 CT number to density and SPR

**FIGURE 4:**
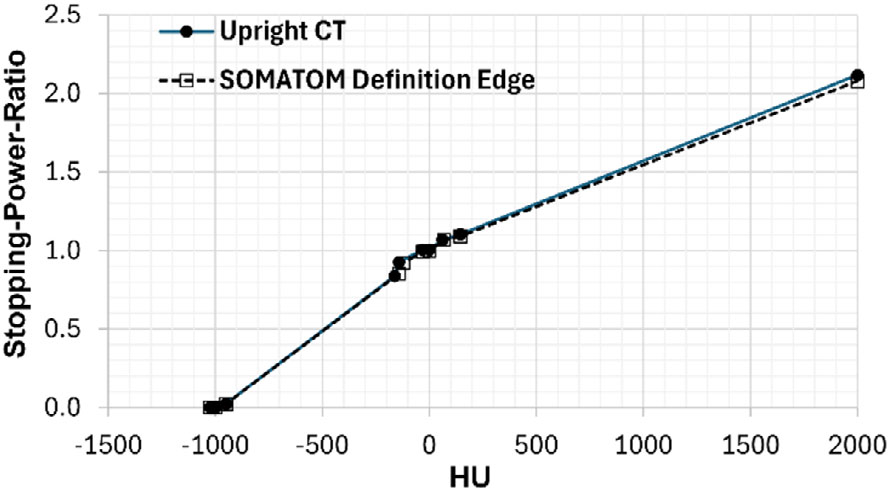
Upright CT number to SPR HLUT.

The upright CT number to SPR calibration closely matched the recumbent CT simulator as shown in Figure 4. The mean absolute HU difference was 28 HU, and the largest discrepancy was 52 HU in bone (1.93 g/cm^3^). CT number linearity versus the reference CT simulator was excellent (R^2^ = 0.9997, Figure S2). An example upright CT image of the Advanced Electron Density Phantom and the HU to mass density calibration are shown in Supplemental Figure S2.

### 3.4 Photon and proton dose calculations

**FIGURE 5:**
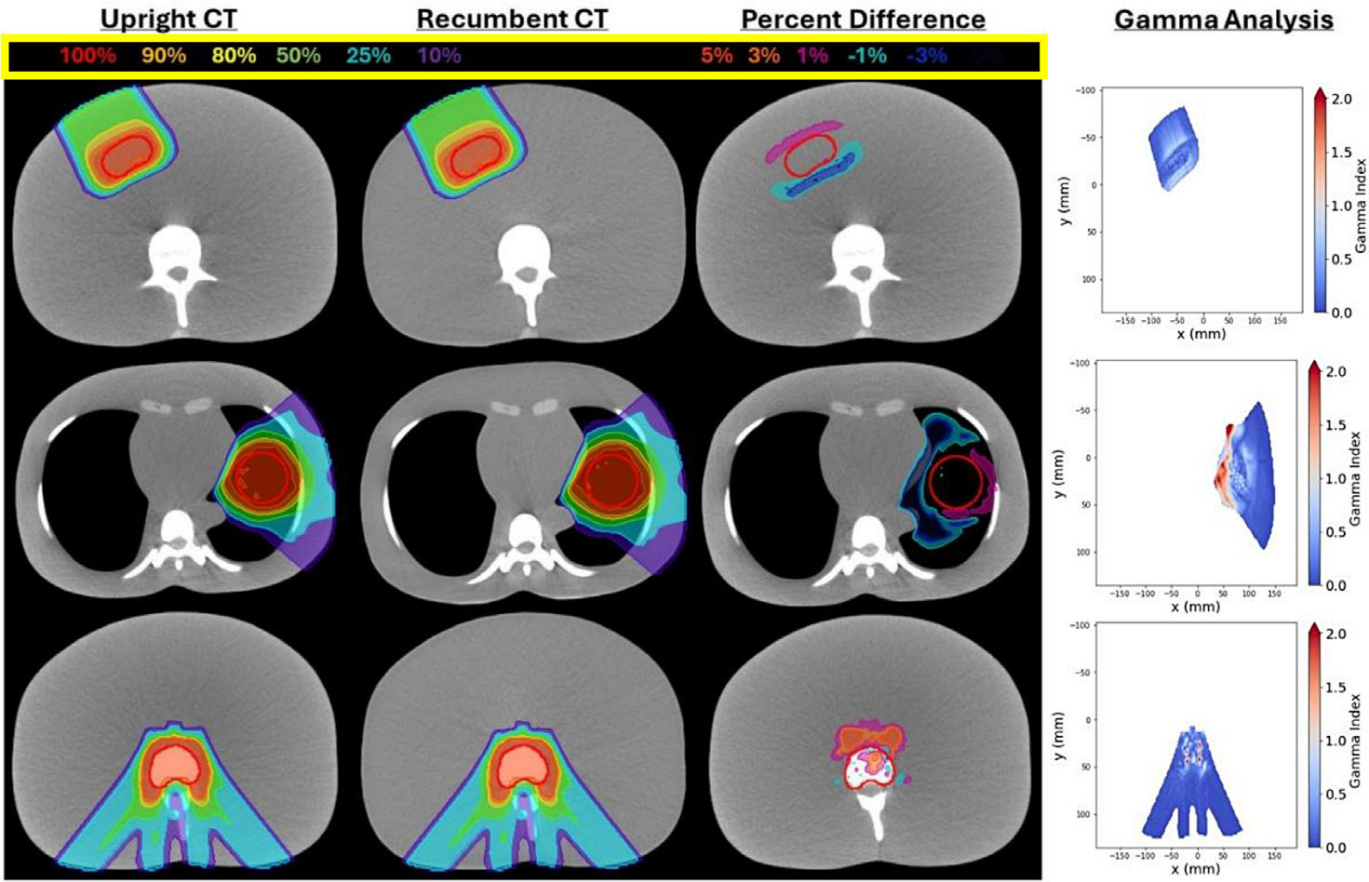
Proton liver (top), lung (middle), and spine (bottom) plans on upright (first column) and registered recumbent CTs (second). Percent dose difference (third) and gamma maps (fourth) show local variations.

Liver, lung, and spine proton dose distributions are presented on upright and recumbent axial CT images in Figure 5. Gamma analysis and percentage dose difference maps are included to highlight regions of greatest disagreement. Additional images in coronal and sagittal views along with photon dose distributions are provided in Figures S3 and S4.

Gamma analysis pass rates are summarized in Table 5. Gamma pass rates were ≥99.8% for all photon treatment plans with 1%/1 mm criteria, demonstrating excellent agreement between dose calculations on upright and recumbent CT datasets. For proton plans, gamma pass rates were ≥97.0% for the spine and liver plans but were slightly lower at 90.6% for the lung plan. All photon and proton plans satisfied the 95% pass rate tolerance recommended by AAPM TG‐218 (3%/2 mm criteria).^21^ Dosimetric consistency between upright and recumbent CT volumes is further supported by Supplemental Figure S5 which presents dose volume histograms for a summed dose distribution of the spine, liver, and lung plans.

**TABLE 5:** Global gamma analysis pass rates are presented for photon and proton dose calculations performed on upright versus recumbent CT acquired images.

	Photon			Proton		
	1%/1 mm	2%/2 mm	3%/2 mm	1%/1 mm	2%/2 mm	3%/2 mm
Liver	100.0%	100.0%	100.0%	99.9%	100.0%	100.0%
Spine	99.8%	100.0%	100.0%	97.0%	99.5%	99.7%
Lung	100.0%	100.0%	100.0%	90.6%	97.7%	98.0%

## 4 DISCUSSION

This work presents the first images acquired on a novel upright CT simulator, demonstrating clinically acceptable image quality and dose calculation accuracy for radiotherapy simulation and treatment planning.

Measured CTDI_vol_ values were comparable to published ranges for recumbent CT simulators. The *Head Hi‐Res Adult* was 23.5 ± 0.02 mGy, within the 50^th^–75^th^ percentile range (20–33 mGy) of head and neck CT doses.^9^ The *Thorax Vendor Default* CTDI_vol_ was 10.1 ± 0.01 mGy, and within the range of 3.9–24.2 mGy for lung protocols.^10^ Measured CTDI values agreed within 10% of the vendor estimate, satisfying the AAPM TG‐66 tolerance of < 20%.^14^, ^24^

Metrics of image quality including high‐contrast spatial resolution, low contrast performance, spatial integrity, CT number accuracy, and image uniformity were assessed using the ACR‐464 phantom. High‐contrast spatial resolution exceeded the ACR tolerance (6 lp/cm) with 8 lp/cm observed. Results were corroborated by MTF analysis. The CNR was 0.93, narrowly below the ACR tolerance of ≥1.0. Only the 25 mm diameter low‐contrast object was visible. Smaller objects were obscured by mild cupping artifacts from image non‐uniformity which measured 20.2 HU (≤7 HU tolerance). CT number accuracy met tolerances for water and acrylic, with minor deviations for air, polyethylene, and bone. The mean CT number σ (2.1 HU) of seven measurements was comparable to monthly measurements on the recumbent CT (1.4 HU). The maximum difference of 27 HU observed for the bone insert would be accounted for in the scanner‐specific CT number to SPR calibration,^15^ supporting accurate conversion of CT number to mass density and SPR. Future work is warranted to refine beam hardening and scatter corrections to improve CT number accuracy to a level that meets ACR standards. In‐plane spatial integrity median error was 0.23 mm, well within the ≤1.0 mm tolerance recommended by the AAPM Task Group 66, and supports the scanner's suitability for treatment planning.^14^

This study demonstrates the initial feasibility of performing accurate photon and proton dose calculations on upright CT images using a heterogeneous anthropomorphic phantom. Gamma pass rates exceeded 90.6% with 1%/1 mm criteria and 98.0% with 3%/2 mm, indicating comparable performance to the recumbent CT baseline under controlled conditions. Future work will include end‐to‐end validation with measurement‐based comparisons after the upright CT system is integrated with a fixed proton beamline. Additional evaluation of artifacts and the dosimetric impact in human subjects is also warranted. A limitation of this study, consistent with current clinical practice, is that all proton dose calculations were based on single‐energy CT and a corresponding CT number‐to‐SPR calibration. Future work incorporating dual‐energy CT derived SPRs may improve accuracy as this technology becomes readily available. Nevertheless, a recent NRG Oncology Survey reported that the majority (88%) of centers continue to rely on single‐energy CT for proton therapy dose calculations rather than dual‐energy CT.^25^

The upright CT system is undergoing a phase of rapid development. As a result, several limitations exist including the calibrated imaging energy at 120 kVp. Efforts to improve low contrast detectability and ring artifacts using more advanced algorithms and anti‐scatter grids are underway. Four dimensional CT, iterative reconstruction, dual energy CT, and metal artifact reduction methods have yet to be implemented on the system. Future work to evaluate CT number accuracy in the extended HU region is warranted if metal artifact reduction becomes available. In the interim, standard clinical guidelines for proton therapy in the presence of metallic implants should be followed by avoiding proton beam entrance through large metal implants when possible and applying material overrides to artifact affected regions to ensure accurate stopping‐power assignment. These approaches are consistent with recently published recommendations and are current best practice on recumbent CT when metal artifact reduction tools are unavailable as well.^26^

## 5 CONCLUSION

This work establishes the feasibility of upright CT for radiotherapy simulation and treatment planning. Image quality met or approached ACR standards, and photon and proton dose calculations agreed closely with conventional recumbent CT results. These findings support the clinical viability of upright CT for radiotherapy applications. Future efforts will integrate the scanner with a fixed proton beamline, validate IGRT workflows, and assess anatomical and dosimetric differences between upright and recumbent positioning to identify disease sites that may benefit most.

## CONFLICT OF INTEREST STATEMENT

John Hayes and Carson Hoffman disclose employment by Leo Cancer Care, Inc. Jessica Miller has research collaboration funding with Siemens Healthineers. Carri Glide‐Hurst reports research collaborations with RaySearch and Leo Cancer Care, Inc. pertaining to the work. Carri Glide‐Hurst reports research collaborations with GE Healthcare and Medscint Inc outside the submitted work. Jordan Slagowski, Yuhao Yan, and Minglei Kang have no conflicts of interest to report.

## Supporting information



Supporting Information
