# Supplementary material for: Technical assessment of a novel vertical CT system for upright radiotherapy simulation and treatment planning
Source: Med Phys. 2026 Feb 13;53(2):e70312. doi: 10.1002/mp.70312 (PMC12904084; doi:10.1002/mp.70312)

**SUPPLEMENTAL DATA**

| **Target or Organ-at-Risk** | **Planning Goal** |
| --- | --- |
| Spine PTV | D95% > 27 Gy (3 fx) |
| Lung | V11.6 Gy < 1500 cm^3^ |
| Lung | V12.4 Gy < 1000 cm^3^ |
| Spinal Cord | D0.03 cm^3^ < 18 Gy |
| Lung PTV | D95% > 74 Gy (37 fx) |
| Esophagus | D0.03 cm^3^ < 80 Gy |
| Spinal Cord | D0.03 cm^3^ < 45 Gy |
| Heart | Mean < 26 Gy |
| Lung | Mean < 20 Gy |
| Lung | V20 Gy < 35% |
| Lung | V10 Gy < 45% |
| Lung | V5 Gy < 65% |
| Liver PTV | D95% > 50 Gy (5 fx) |
| Spinal Cord | D0.03 cm^3^ < 22 Gy |

**Table S1:** Treatment planning goals.

**Table 1:** Treatment planning goals.

**Figure S1:** An upright CT image [-300 HU, 100 HU] of a 48 cm diameter Helios IQ Cal phantom is shown with horizontal and vertical line profiles.

**Figure 1Figure 2:** An upright CT image [-300 HU, 100 HU] of a 48 cm diameter Helios IQ Cal phantom is shown with horizontal and vertical line profiles.


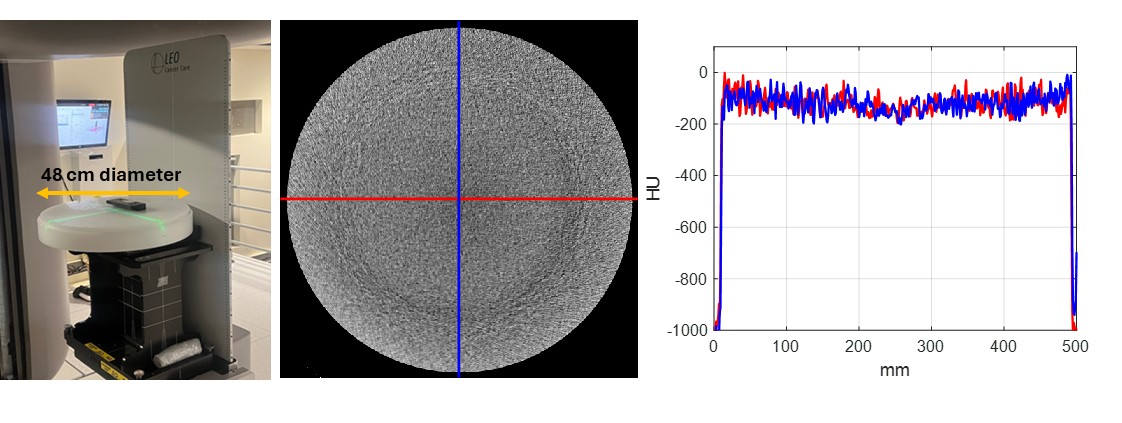


**Figure S2:** (A) An upright CT image of the CT number to electron density phantom is shown [WL = 0 HU, WW = 1000 HU]. (B) Upright CT number linearity is evaluated versus a reference CT scanner (R^2^=0.9997). CT number versus mass density (C) is shown for the upright CT scanner versus a recumbent CT system.


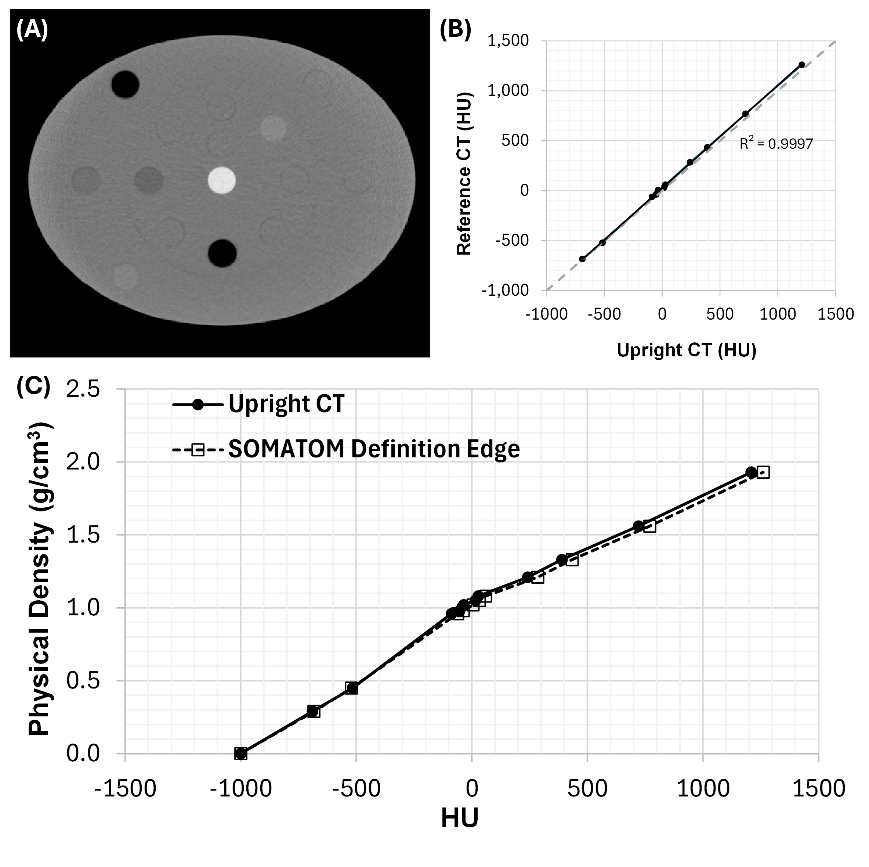


**Figure S3:** Upright and recumbent CT images of an anthropomorphic thorax phantom with proton doses are presented for soft tissue [40, 350] HU, lung [-600, 1600] HU, and bone [450, 1600] HU windows and levels.

**Table 2Figure 3:** Upright and recumbent CT images of an anthropomorphic thorax phantom with proton doses are presented for soft tissue [40, 350] HU, lung [-600, 1600] HU, and bone [450, 1600] HU windows and levels.


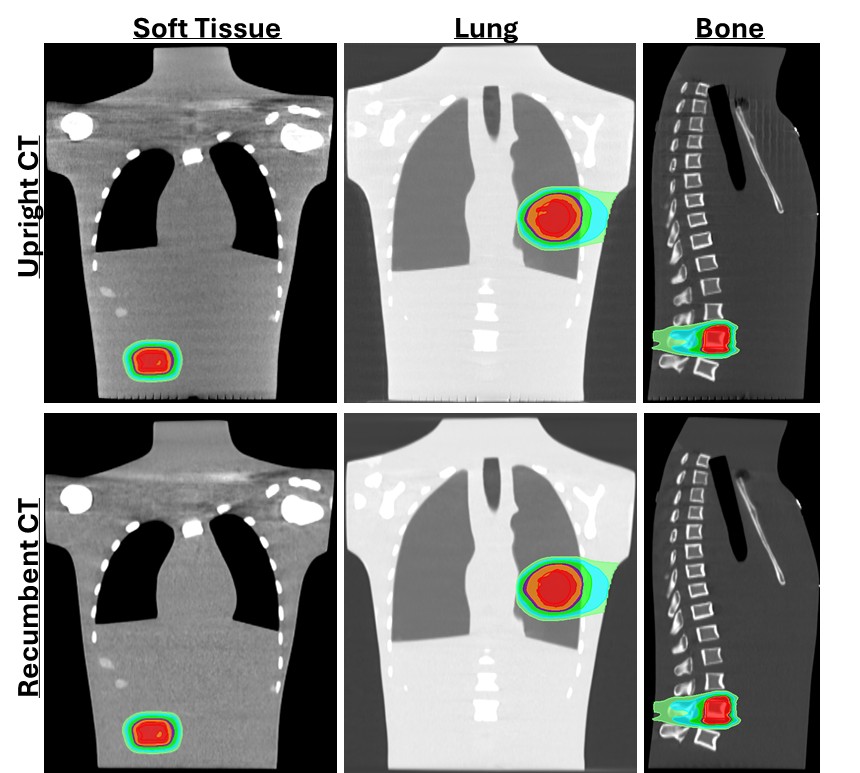


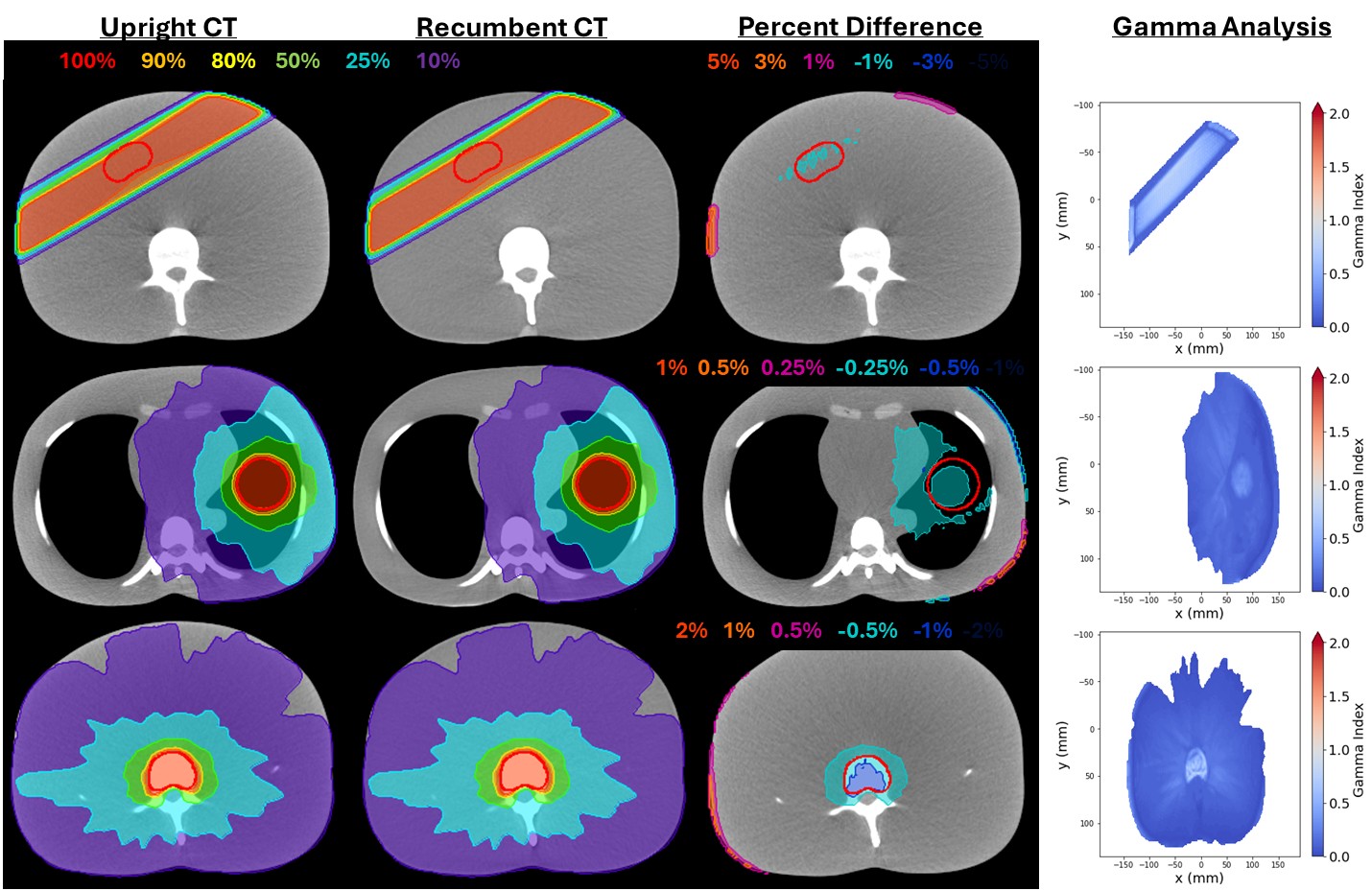


**Figure S4:** Photon liver (top row), lung (middle row), and spine (bottom row) plans optimized and calculated on upright CT images are presented (first column) versus dose re-computed on registered recumbent CT images (second column). Percent dose difference (third column) and gamma analysis maps (fourth column) highlight local differences.

**Figure 4Figure 5:** Photon liver (top row), lung (middle row), and spine (bottom row) plans optimized and calculated on upright CT images are presented (first column) versus dose re-computed on registered recumbent CT images (second column). Percent dose difference (third column) and gamma analysis maps (fourth column) highlight local differences.


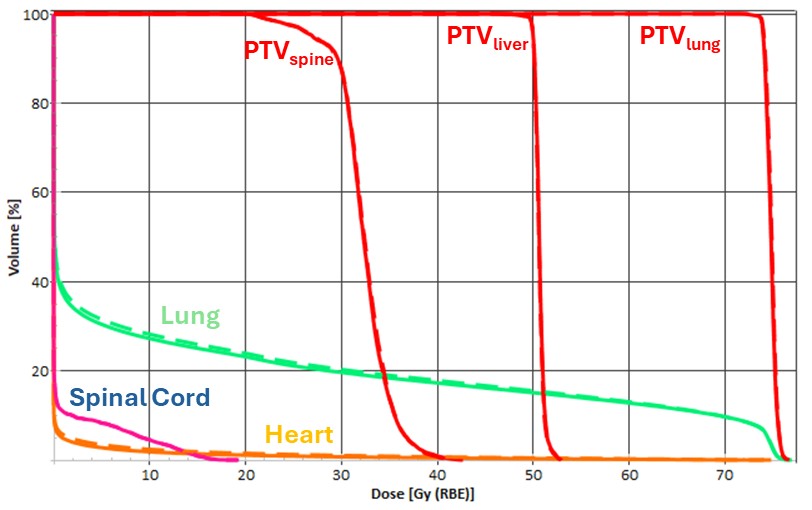


**Figure S5:** Dose volume histograms for summed spine, liver, and lung plans are shown for protons (top) and photons (bottom). Solid lines represent dose computed on upright CT images; dashed lines represent dose on recumbent CT images.

**Figure 6:** Dose volume histograms for summed spine, liver, and lung plans are shown for protons (top) and photons (bottom). Solid lines represent dose computed on upright CT images; dashed lines represent dose on recumbent CT images.


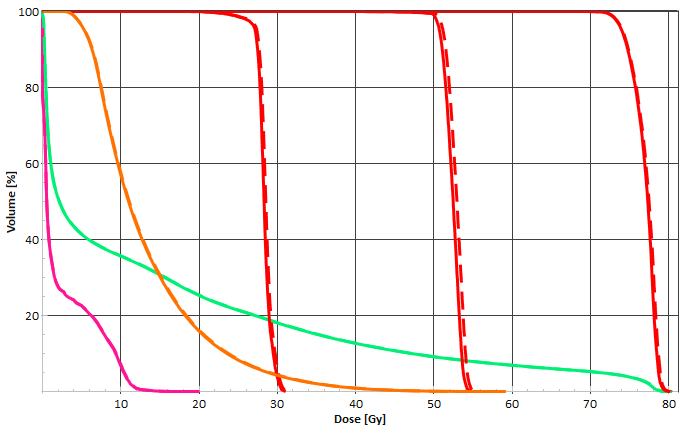

Supplement: Supplementary file 1 — Supporting Information [file MP-53-0-s001.docx]
